# Prognostic Relevance of 18F-FDG-PET/CT-Guided Target Volume Delineation in Loco-Regionally Advanced Nasopharyngeal Carcinomas: A Comparative Study

**DOI:** 10.3389/fonc.2021.709622

**Published:** 2021-08-23

**Authors:** Ouying Yan, Hui Wang, Yaqian Han, Shengnan Fu, Yanzhu Chen, Feng Liu

**Affiliations:** ^1^Department of Radiation Oncology, Hunan Cancer Hospital and The Affiliated Cancer Hospital of Xiangya School of Medicine, Central South University, Changsha, China; ^2^The Affiliated Cancer Hospital of Xiangya School of Medicine, Central South University, Changsha, China

**Keywords:** FDG-PET/CT, prognosis, nasopharyngeal carcinoma, chemoradiotherapy, intensity-modulated radiation therapy

## Abstract

**Introduction:**

An optimal approach to define tumor volume in locoregionally advanced nasopharyngeal carcinoma (NPC) using 18F-fluorodeoxyglucose positron emission tomography/computed tomography (FDG-PET/CT) remains unclear. This retrospective study aimed at comparing the outcomes and toxicities of different FDG-PET/CT-guided techniques for primary tumor volume delineation in locoregionally advanced NPC.

**Methods:**

From August 2015 to February 2018, 292 patients with stage III-IVB NPC received FDG-PET/CT-guided IMRT. Three PET/CT-based techniques were used to determine the gross tumor volume (GTV) as follows: visual criteria (group A; n = 98), a standard uptake value (SUV) threshold of 2.5 (group B; n = 95), and a threshold of 50% maximal intensity (group C, n = 99) combined with a dose-painting technique.

**Results:**

In groups A, B, and C, the 5-year LRFS rates were 89.4%, 90.0%, and 97.8%, respectively (p = 0.043). The 5-year DMFS rates were 75.1%, 76.0%, and 87.7%, respectively (p = 0.043). The 5-year DFS rates were 70.9%, 70.3%, and 82.2%, respectively (p = 0.048). The 5-year OS rates were 73.5%, 73.9%, and 84.9%, respectively (p = 0.038). Group C showed significantly higher 5-year LRFS, LRRFS, DMFS, DFS, and OS than those in groups A and B (p < 0.05). No statistically significant differences were observed between the three study groups in the cumulative incidences of grade 3-4 acute and late toxicities. Multivariate analyses showed that the PET/CT-guided technique for target volume delineation was an independent prognostic factor for 5-year LRFS, DFS, DMFS, and OS (p = 0.039, p = 0.030, p = 0.035 and p = 0.028, respectively), and was marginally significant in predicting LRRFS (p = 0.080).

**Conclusions:**

The 50% SUVmax threshold regimen for GTV delineation with dose-painting appeared to be superior to the visual criteria or SUV2.5 threshold in locoregionally advanced NPC, and there was no increased toxicity.

## Introduction

Nasopharyngeal carcinoma (NPC) is a radiosensitive neoplasm. Radiotherapy (RT) is the primary treatment strategy for NPC, and concurrent chemoradiotherapy is extensively used for locally advanced NPC (1, 2). However, the treatment response is unsatisfactory, with rates of local recurrence varying from 16.8% to 23% (3, 4). Since the mortality rate associated with NPC is directly related to the rates of local recurrence, it is important to develop methods for the improvement of treatment outcomes in patients with locoregionally advanced disease. Boosting the radiotherapy dose can provide better local control. However, dose escalation for NPC may increase treatment-related comorbidities due to the high-dose irradiation of normal tissues (5). Thus, determining the appropriate tumor volume to prescribe high radiation dose treatment remains a major challenge.

18F-fluorodeoxyglucose positron emission tomography/computed tomography (FDG-PET-CT) is a powerful molecular imaging tool based on the activity of cancer cell metabolism. Delineation of biological characteristics prior to the therapy facilitates individual adaptation and optimization of treatment schedules and ensures improved prognosis and decreased treatment toxicity (6). Previous studies have indicate that 18F-FDG PET can be used for target volume delineation in radiotherapy for head and neck squamous cell carcinomas (including NPC) (3, 6–13). Several approaches have been proposed for outlining FDG-avid tumors, including auto-contouring at SUV threshold ≥ 2.5, ≥ 40% to 50% of maximal SUV (SUVmax) and visual delineation (14). In our previous study, we compared FDG-PET/CT guided dose escalation IMRT with CT-based IMRT in locoregionally advanced NPC. Relative to CT-based IMRT, FDG-PET/CT-guided dose-painting IMRT (DP-IMRT) is a powerful technique with survival benefit which does not increase the incidence of toxicities (3).

To the best of our knowledge, the methods and thresholds based on SUV have not been clearly defined till date. Additionally, clinical trials directly comparing the long-term results of IMRT based on different PET/CT-derived GTV delineation in NPC patients are not available. The primary aim of this study was to retrospectively analyze the comparative efficacy and toxicity of PET/CT-guided IMRT using three PET/CT-derived methods for primary tumor volume delineation in locoregionally advanced NPC patients, and to determine if there was a difference between PET/CT-guided dose-painting and PET/CT-based IMRT in locally advanced NPC prognosis.

## Methods

### Patient Selection

Between August 2015 and February 2018, 292 patients with locoregionally advanced NPC from the Hunan Cancer Hospital (The Affiliated Cancer Hospital of Xiangya School of Medicine, Central South University) were selected for the present study. Eligible patients between the ages of 18-70 years with non-distant metastatic, histologically confirmed WHO types II-III, stage III, and IVB nasopharyngeal carcinoma. Patients were required to provide written informed consent prior to undergoing chemoradiotherapy. Patients with a history of previous radiotherapy, in-complete radiotherapy, secondary malignancy, evidence of distant metastasis, pregnancy, or lactating females were excluded from the study. We were able to identify the information of participants during and after data collection. This retrospective study was approved by the Ethics Committee of our hospital.

### Radiotherapy

All patients received both pre-treatment contrast-enhanced CT of the head and neck and 18F-FDG-PET/CT of the whole body. The scope of the CT simulation scan from the head to the manubriosternal joint was at 2.5-mm increments. The FDG-PET/CT scans were performed within 3 days of CT scans of the same location and in same the postural position. At 1-hour post-injection of 190-240 MBq of FDG, FDG-PET scans were conducted. Data acquisition was within 3 minutes per bed position (3, 9). The images were then converted from FDG-PET to SUV, and PET/CT and CT images were used for image fusion. Three FDG-PET/CT-based methods for gross tumor volume (GTV) delineation were compared: visual criteria (group A), a standard uptake value (SUV) threshold of 2.5, (SUV2.5) (group B), and a threshold of 50% of the maximum standardized uptake value (50% SUVmax) (group C), combined with dose-painting technique. The target volumes were based on FDG-PET/CT by a group of experienced radiation oncologists, with the assistance of experienced nuclear medicine physicians. In group A, the criteria for defining the GTV of the nasopharynx (GTVnx) in FDG-PET/CT were based on visual observation (volume) (10, 12, 13). In group B, the primary tumor area with SUV2.5 threshold was defined as GTVnx (volume 2.5) (8, 11, 14). In group C, the visual criteria were used for GTVnx delineation. Using a dose-painting technique for simultaneous integrated boost (SIB), a sub-volume GTVnx-PET (volume 50%) in the GTVnx was defined as the 50% threshold of the maximum standardized uptake value (3, 9, 11, 14, 15).

In all the groups, IMRT was performed using linear accelerators (16–18). The GTVnx was enlarged by 5 mm (containing the whole nasopharyngeal mucosa and submembrane) (17), and defined as PGTVnx. The dose for T1-2 patients was DT 70.4 Gy/32 Fx, and for T3-4 patients was DT 72.6 Gy/33 Fx, with 2.2 Gy per fraction. The irradiation doses of lymph node GTV (GTVnd) was 69.96-72.6 Gy/32-33 Fx, with 2.12-2.2 Gy per fraction; for high-risk subclinical lesions (planned target volume, PTV1), it was 60.06-64 Gy/32-33 Fx, with 1.82-2.0 Gy per fraction, and for low-risk subclinical diseases (PTV2) it was 50.96-56.0 Gy/26-28 Fx, at the rate of 1.82-2.0 Gy per fraction. Radiotherapy was performed daily from Monday to Friday and lasted for 32 to 33 days. The Pinnacle3 inverse planning system was used to design and optimize the regimens. Group C was subjected to PET/CT-guided DP-IMRT. The dose administered to the GTVnx-PET was increased to DT 75.2 Gy/32 Fx gradually in T1-2 patients, and DT 77.55 Gy/33 Fx in T3-4 patients, at 2.35 Gy per fraction. Other dose target volumes were prescribed in a manner similar to those in groups A and B. The doses of critical structures were within the tolerance limits of the Radiation Therapy Oncology Group (RTOG) 0615 (16) and RTOG 0225 protocols (18).

### Chemotherapy

Induction chemotherapy was administered every 3 weeks, which consisted of intravenous 3 cycles of docetaxel (60 mg/m2) and cisplatin (60 mg/m2) on day 1, followed by uninterrupted intravenous fluorouracil administration (600 mg/m2) per day from day 1 to day 5, for three cycles before concurrent chemoradiotherapy. The prescription of concurrent chemotherapy was 80-100 mg/m2 cisplatin alone every three weeks, at the same time as IMRT.

### Follow-Up

The follow-up period was calculated from day one of the therapy through the last date of follow-up (April 16, 2021) or until death. We classified chemotherapy-related toxicities based on the Common Terminology Criteria for Adverse Events (version 4.0) and evaluated the toxicities of radiotherapy based on the RTOG scoring criteria for acute and late radiation incidences. The tumor complete response (CR) was assessed by physical examination of the head and neck, fiberoptic nasopharyngoscopy, and MRI at 3 months after radiotherapy completion. Classification of tumor response was based on WHO response standard (16, 19).

### Statistical Analysis

All analyses were performed using SPSS (version 20.0; IBM Corporation, Armonk, NY, USA). The overall survival (OS) was defined as the time from diagnosis to the last available follow-up; disease-free survival (DFS), survival without any local, regional, or distant failure; distant metastasis-free survival (DMFS), as survival without distant metastasis; local recurrence-free survival (LRFS), as survival without local relapse; regional recurrence-free survival (RRFS), survival without local relapse in cervical or regional lymph nodes, and locoregional recurrence-free survival (LRRFS), as survival without local relapse in the lymph nodes of the nasopharynx or cervical.

The classification variables were analyzed using χ² test. Kaplan-Meier survival curves and log-rank tests were used to calculate time-to-event endpoints between the three groups. Multivariable analyses were performed to assess the significance of independent prognosis using the Cox proportional hazards model. The potential prognostic factors included age, sex, tumor stage, node stage, pre-treatment Epstein-Barr virus deoxyribonucleic acid (EBV DNA) concentration (20), and PET/CT-guided GTV (50% SUVmax threshold *vs*. visual criteria or SUV2.5 threshold). Statistical significance was set at p<0.05.

## Results

### Patient Characteristics

The number of patients in groups A, B, and C were 98, 95, and 99, respectively. The median age was 47 years (range: 18-70 years). The median follow-up time for all patients was 60.5 months (range: 13-68 months) and 62 months for the surviving patients (range: 39-68 months). The median SUVmax value for nasopharyngeal masses was 10.6 (range: 4.2-25.3) for all patients. The patient baseline features are listed in **Table 1**. Clinical features and baseline demographics were balanced between the three groups.

**Table 1:** Clinical demographics of patients with loco-regionally advanced NPC.

Characteristics	Visual criteria group	SUV 2.5 group	50% SUV max group	P value*
	No. of patients (%)	No. of patients (%)	No. of patients (%)	
Total	98	95	99	
Age, y				
Median	47	47	46	
Range	18-66	19-69	22-70	
Sex				
Male	71 (72.4)	68 (71.6)	67 (67.7)	0.736
Female	27 (27.6)	27 (28.4)	32 (32.3)	
Pathology				
WHO type 2	30 (30.6)	30 (31.6)	29 (29.3)	0.941
WHO type 3	68 (69.4)	65 (68.4)	70 (70.7)	
T stage				
T1	11 (11.2)	10 (10.5)	9 (9.1)	0.989
T2	25 (25.5)	24 (25.3)	27 (27.3)	
T3	28 (28.6)	25 (26.3)	30 (30.3)	
T4	34 (34.7)	36 (37.9)	33 (33.3)	
N stage				
N0	3 (3.1)	3 (3.2)	3 (3.0)	0.974
N1	6 (6.1)	7 (7.4)	10 (10.1)	
N2	68 (69.4)	63 (66.3)	65 (65.7)	
N3	21 (21.4)	22 (23.2)	21 (21.2)	
AJCC stage group				
III	52 (53.0)	47 (49.5)	48 (48.5)	0.971
IVA	27 (27.6)	28 (29.5)	31 (31.3)	
IVB	19 (19.4)	20 (21.1)	20 (20.2)	
Concurrent chemotherapy				
Yes	98 (100.0)	95 (100.0)	99 (100.0)	.
No	0 (0.0)	0 (0.0)	0 (0.0)	
Induction chemotherapy				
Yes	23 (23.5)	26 (27.4)	22 (22.2)	0.686
No	75 (76.5)	69 (72.6)	77 (77.8)	
Adjuvant chemotherapy				
Yes	11 (11.2)	10 (10.5)	8 (8.1)	0.741
No	87 (88.8)	85 (89.5)	91 (91.9)	

*P values were calculated using chi-square test.

### Impact of PET/CT-Derived GTV Delineation on Primary Tumor Volume

The median primary tumor volumes of the GTVnx for group A (visual volume), group B (volume 2.5), and group C were 41.9 mL (range: 6.2-184.6 mL), 36.5 mL (range: 4.6-162.2 mL), and 39.4 mL (range: 5.8-176.8 mL), respectively. The median volume of the GTVnx-PET (volume 50%) in group C was 13.2 mL (range: 1.4-32.6 mL). The volume 50% in group C was significantly lower than the visual volume in group A (p < 0.001), volume 2.5 in group B (p < 0.001), and GTVnx in group C (p < 0.001). No significant differences were found in GTVnx for the three groups (group A *vs*. group B, p = 0.056; group A *vs*. group C, p = 0.141; group B *vs*. group C, p = 0.704).

### Response

The complete response (CR) rate in group C was 100% (99/99) as compared to 92.9% (91/98) in group A (p = 0.007) and 94.7% (90/95) in group B (p = 0.021). The CR rate did not differ significantly between groups A and B (p = 0.607). Two patients in group A and one in group B showed residual nasopharyngeal tumors. Six patients in group A and four in group B showed residual neck lymph nodes. Three patients had residual nasopharyngeal neoplasms received salvage chemotherapy. Six months after the completion of radiotherapy, no patient with residual nasopharyngeal tumors was observed and only 2 patients in group A and 1 patient in group B were diagnosed with residual neck lymph nodes, and were successfully treated with salvage neck dissection.

### Adverse Events

All patients in the cohorts completed the established RT regimen. All patients received concurrent chemotherapy. 71 patients (24.3%) underwent induction chemotherapy. 29 (9.9%) patients received adjuvant chemotherapy after completion of radiotherapy (**Table 1**). Chemotherapy was discontinued due to severe liver dysfunction, neutropenia, and refusal of treatment. Mucositis and hematologic toxicity were the most frequently recorded grade 3-4 acute adverse events. Three patients experienced grade 3-4 late toxicities, including skin fibrosis and xerostomia (dry mouth) (**Table 2**). No treatment-related deaths occurred during treatment. Tumor responses and toxicities were similar among the three groups (**Table 2**).

**Table 2:** Grade 3-4 toxicity.

Adverse events	Visual criteria group	SUV 2.5 group	50% SUV max group	P value*
	No. of patients (%)	No. of patients (%)	No. of patients (%)	
Acute adverse events				
Anemia	2 (2.0)	1 (1.1)	1 (1.0)	0.788
Neutropenia	8 (8.2)	10 (10.5)	8 (8.1)	0.796
Leukopenia	18 (18.4)	15 (15.8)	15 (15.2)	0.813
Thrombocytopenia	2 (2.0)	0	1 (1.0)	0.372
Liver dysfunction	1 (1.0)	0	0	0.370
Nephrotoxicity	0	0	0	.
Nausea	17 (17.3)	16 (16.8)	14 (14.1)	0.805
Vomiting	11 (11.2)	13 (13.7)	10 (10.1)	0.730
Mucositis	34 (34.7)	28 (29.5)	31 (31.3)	0.731
Dermatitis	12 (12.2)	12 (12.6)	9 (9.1)	0.692
Dysphagia or odynophagia	4 (4.1)	3 (3.2)	3 (3.0)	0.907
Dry mouth	5 (5.1)	5 (5.3)	3 (3.0)	0.700
Ototoxicity	1 (1.0)	0	0	0.370
Late adverse events				
Skin fibrosis	1 (1.0)	1 (1.0)	0	0.600
Dry mouth	1 (1.0)	0	0	0.370
Ototoxicity	0	0	0	.
Trismus	0	0	0	.
Nasopharyngeal ulceration	0	0	0	.

*P values were calculated using chi-square test.

### Treatment Failure

24 patients (24.5%) in group A, 22 patients (23.2%) in group B, and 12 patients (12.5%) in group C had tumor recurrence. The median time to recurrence was 25.5 (8–38) months for local recurrence, 26 (8–42) months for regional recurrence and was 29 (8–42) months for loco-regional recurrence. 52 patients experienced metastases to distant organs, of whom 46 had bone metastases, 18 had liver metastases, and 30 had lung metastases. 32 patients had more than one metastatic site. In conformity with standard practice, salvage treatments were conducted for the patients with relapse, involving re-irradiation, chemotherapy, and surgery.

### Survival

**Figure 1:**
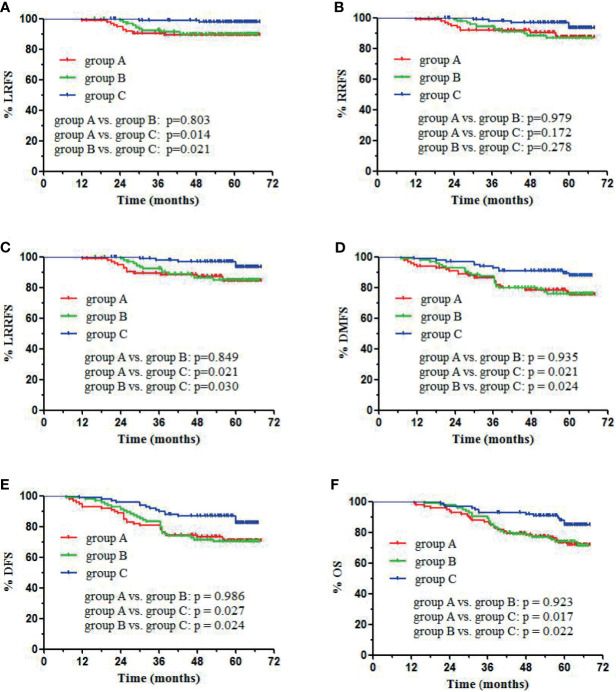
Kaplan-Meier survival curves of different groups: visual criteria, SUV2.5, and 50% SUVmax group. **(A)** LRFS, **(B)** RRFS, **(C)** LRRFS, **(D)** DMFS, **(E)** DFS, **(F)** OS.

64 patients (26 in group A, 25 in group B, and 13 in group C) died, which included 48 deaths due to distant metastases, 9 due to loco-regional recurrence, and 7 due to other medical conditions. In groups A, B, and C, the 5-year LRFS rates were 89.4%, 90.0%, and 97.8%, respectively (p = 0.043). The 5-year RRFS rates were 87.3%, 87.0%, and 93.4%, respectively (p = 0.170). The 5-year LRRFS rates were 84.3%, 84.9%, and 93.4%, respectively (p = 0.054). The 5-year DMFS rates were 75.1%, 76.0%, and 87.7%, respectively (p = 0.043). The 5-year DFS rates were 70.9%, 70.3%, and 82.2%, respectively (p = 0.048), and the 5-year OS rates were 73.5%, 73.9%, and 84.9%, respectively (p = 0.038). No statistically significant differences in LRFS, RRFS, LRRFS, DMFS, DFS, and OS were observed between groups A and B (**Figure 1**). Group C showed significantly higher 5-year LRFS, LRRFS, DMFS, DFS, and OS (p < 0.05, **Figure 1**) as compared with group A or group B.

### Prognostic Factors

In the univariate analysis, the PET/CT-guided GTV (50% SUVmax threshold *vs*. visual criteria or SUV2.5 threshold) was an important prognostic factor for 5-year LRFS, LRRFS, DMFS, DFS, and OS (p = 0.013, p = 0.016, p= 0.012, p= 0.014, and p = 0.011, respectively). EBV DNA was identified as an important prognostic factor for 5-year LRFS, RRFS, LRRFS, DMFS, DFS, and OS (p < 0.001, p = 0.017, p = 0.003, p < 0.001, p < 0.001, and p < 0.001, respectively). There was a significant correlation between sex and DMFS (p = 0.035). However, age, T-category, and N-category were not significant factors for LRFS, RRFS, LRRFS, DMFS, DFS, or OS. Multivariate analyses revealed that PET/CT-guided GTV was an independent prognostic indicator of 5-year LRFS, DFS, DMFS, and OS (p = 0.039, p = 0.030, p = 0.035 and p = 0.028, respectively), and was marginally significant for LRRFS (p = 0.080). EBV DNA was a favorable independent prognostic indicator of 5-year LRFS, LRRFS, DFS, DMFS, and OS (p = 0.010, p = 0.043, p < 0.001, p< 0.001, and p < 0.001, respectively). The outcomes from the multivariate Cox regression analyses are listed in **Table 3**.

**Table 3:** Multivariable analysis of prognostic factors in loco-regionally advanced NPC.

Endpoint	HR (95% CI)	P value*
Local recurrence-free survival		
Sex	0.631 (0.254-1.570)	0.322
Age	1.350 (0.565-3.225)	0.500
T stage	1.001 (0.401-2.501)	0.998
N stage	0.000 (0.000-0.000)	0.977
EBV DNA level	0.070 (0.009-0.526)	0.010
PET-guided GTV	4.655 (1.081-20.047)	0.039
Loco-regional recurrence-free survival		
Sex	0.657 (0.293-1.472)	0.308
Age	1.094 (0.503-2.379)	0.820
T stage	0.819 (0.350-1.920)	0.646
N stage	0.369 (0.049-2.802)	0.335
EBV DNA level	0.381 (0.150-0.968)	0.043
PET-guided GTV	2.392 (0.900-6.359)	0.080
Disease-free survival		
Sex	1.175 (0.672-2.063)	0.572
Age	1.410 (0.885-2.249)	0.149
T stage	1.216 (0.737-2.008)	0.445
N stage	1.047 (0.441-2.488)	0.917
EBV DNA level	0.251 (0.136-0.464)	<0.001
PET-guided GTV	1.863 (1.063-3.264)	0.030
Distant metastasis-free survival		
Sex	1.935 (0.912-4.107)	0.086
Age	1.566 (0.920-2.666)	0.099
T stage	1.139 (0.638-2.033)	0.086
N stage	1.131 (0.437-2.930)	0.099
EBV DNA level	0.230 (0.111-0.478)	<0.001
PET-guided GTV	2.047 (1.051-3.986)	0.035
Overall survival		
Sex	1.190 (0.656-2.156)	0.567
Age	1.258 (0.767-2.064)	0.363
T stage	1.225 (0.721-2.079)	0.453
N stage	0.751 (0.266-2.119)	0.588
EBV DNA level	0.217 (0.109-0.432)	<0.001
PET-guided GTV	1.988 (1.077-3.668)	0.028

*P values were calculated using an adjusted Cox proportional-hazards model.

## Discussion

Chemoradiotherapy is the primary treatment for locoregionally advanced NPCs. Its clinical outcomes have greatly improved with IMRT (21–23). However, residual tumor and local recurrence are challenging because of the highly invasive and metastatic nature of the disease (3, 4, 23). During IMRT planning, the precise definition of tumor volume is crucial for predicting patient prognosis. Usually, the GTV in NPC is evaluated using CT imaging. However, previous studies have found that 18F-FDG-PET/CT can greatly enhance the value of TNM staging, treatment assessment, and prognosis of NPC (24–28), and has been increasingly used to plan RT (29). Moreover, 18F-FDG-PET integrated with IMRT is more likely to facilitate target volume delineation and dose escalation (30), thereby being more favorable for the main clinical outcomes.

SUV is the primary quantitative indicator for tumor detection using 18F-FDG-PET (9). Several methods for tumor delineation using FDG-PET have been studied. A simple and most commonly used strategy is based on the visual interpretation of FDG-PET images by practiced radiation oncologists or nuclear medicine physicians (10, 12, 13). However, visual delineation of neoplasms is highly operator-dependent and leads to significant inter-observer differences (14). Other techniques of threshold determination have also been used to define target volumes, such as the percentage of maximum peak SUV (50% SUVmax), a fixed SUV threshold of 2.5, or a threshold that is adaptive to the signal-to-background ratio (SBR), although their prognostic value remains controversial (8–11, 14, 15). The lack of a unified standardization technique poses a major challenge in using FDG-PET in delineating tumor volume. Currently, there is no formally recognized method for defining the optimal tumor volume using FDG-PET/CT. Therefore, our research team conducted the current study to ascertain the optimal SUV-based methods to define the primary tumor volume in locoregionally advanced NPC and to compare the difference between PET/CT-guided dose escalation and PET/CT-based (without dose escalation) IMRT for locally advanced NPC prognosis.

Previous studies have combined FDG-PET (or PET/CT) with RT planning and compared the major neoplasm volume using PET and CT (and/or MRI) in patients with NPC (8, 10, 14, 15, 31, 32). Most results showed significant variations between the different modalities. Hung et al. (14) compared different PET-based thresholds (for e.g., SUV2.5, 40% Max, and 50% Max) for primary tumor delineation in 32 NPC patients, and reported that the SUV2.5 method generated the largest volume and the 50% Max method resulted in the smallest tumor volume. In our study, no significant difference was observed between the visual volume and volume 2.5 groups. The primary neoplasm volumes evaluated using the visual and SUV2.5 methods were significantly larger than those derived from the 50% SUVmax method, which is consistent with the findings of Hung et al. (14).

Yu et al. (11) reported that since the volume based SUV50%max isocontour was significantly smaller than the volume derived from the SUV2.5 threshold, the areas of 50% SUVmax may not be sufficient for GTVnx. Therefore, we used similar visual criteria to define GTVnx in the 50% SUVmax group and used dose-painting technique to dose boost for the threshold of 50% SUVmax (GTVnx-PET), which based on our previous study and ongoing clinical trials (9, 15). However, larger target volumes may result in higher doses of irradiation to normal tissues and, thus, increase treatment-related complications. Therefore, our study did not escalate the dose to the PET target volume based on the SUV2.5 threshold and visual delineation.

All patients in our study received concurrent chemoradiotherapy. The CR rate after chemoradiotherapy for locoregionally advanced NPC has been reported to range from 82.8% to 99% (1, 20, 33). We previously reported that FDG-PET/CT-guided DP-IMRT significantly advanced CR rates (99.0%) compared with those by the CT-based IMRT (92.9%) (3). In the present study, the CR rate was significantly higher in group C (100%) than in group A (92.9%) and group B (94.7%). Our results suggest that the risk of local residual tumor was reduced by DP-IMRT using dose escalation to the 50% SUVmax sub-volume.

Very few studies have used PET/CT-guided IMRT to study the nasopharyngeal carcinoma. Wang et al. (8) included 67 patients with locally advanced NPC and analyzed the results of conventional RT, CT-based IMRT, and PET/CT-guided IMRT. The PET/CT-guided IMRT group, which used the SUV2.5 method for delineating GTV, when statistically compared with the traditional RT group, showed a better 3-year local progression-free survival rate (LPFS: 100% *vs*. 95.8%, P<0.05) and DFS (95.2% *vs*. 79.2%, P<0.05). However, the difference in the survival rate between the PET/CT-guided IMRT and CT-based IMRT groups was not statistically significant. We have previously demonstrated that FDG-PET/CT-guided DP-IMRT increased 3-year OS, DFS, DMFS, LFFS, and LRFFS in comparison to the CT-based IMRT (3). Based on these results, we compared the efficacy of PET/CT-guided IMRT using three PET/CT-derived methods for primary tumor volume delineation in patients with locoregionally advanced NPC in the present study. The results showed that the 5-year LRFS, DMFS, DFS, and OS were higher in the 50%SUVmax group than in the visual and SUV2.5 groups. In the current study, dose escalation of the SUV 50%max isocontour improved the treatment efficacy while decreasing collateral damage in comparison to that of the visual criteria and threshold of SUV2.5. Distant metastasis is the primary cause of treatment failure following chemoradiotherapy. Our results suggest that the risk of distant metastasis was highly reduced with an increase in local control rates and, hence, enhanced the DFS and OS. We had a relatively large sample size; thus, the results of our analysis have some instructive significance.

Wang et al. (8) reported that for PET/CT-guided IMRT, the most common acute toxicities included acute mucositis and late toxicities included xerostomia, subcutaneous fibrosis, and ototoxicity. The patients treated with PET/CT-guided or CT-based IMRT showed similar acute and late toxicities. In our study, a single (1.0%) patient with extensive tumor migration to the unilateral parotid gland and metastatic lymph node invasion to the contralateral parotid gland had grade 3 xerostomia in the visual criteria group. To ensure that the dose delivered to the PTV could sufficiently control the tumor, the mean dose of the bilateral parotid gland was increased to 34 Gy in this patient. Bakst et al. (5) evaluated the results of 25 NPC patients (stage II-IVB) who received DP-IMRT combined with chemotherapy. The prescription dose in their trial was 70.2 Gy in 2.34-Gy fractions to the GTV. One patient developed hearing loss of grade 3, and 12% of the patients experienced temporal lobe necrosis. In our study, no patient developed severe ototoxicity or brain toxicity. Compared to the study by Bakst et al., the lower incidence of brain toxicity in our study was likely attributed to a lower fractionated dose in the visual and SUV2.5 groups, and the 50% SUVmax group showed overall smaller dose-escalation volumes. Our PET/CT-based-IMRT regimen did not increase acute and late toxicities in comparison to the CT-based IMRT in NPC patients reported by Lin et al. (17) and Lee et al. (16). Our previous studies found no statistically significant differences in acute and late-presenting toxicities between CT-based IMRT and PET/CT-guided DP-IMRT (3). Likewise, in this study, we did not observe significant differences in acute or late toxicities among the three groups, and no grade 5 acute toxicities were found, which is consistent with prior studies.

Several studies have reported varying levels of prognosis in NPC treated with IMRT combined with chemotherapy (1, 3, 17, 20, 22, 33, 34). However, to date, no prior research has investigated the prognostic value of different techniques in the PET/CT-guided GTV delineation of NPC. Our data showed that PET/CT-guided IMRT (50% SUVmax threshold with dose escalation *vs*. visual criteria or SUV2.5 threshold) was a significant and independent prognostic factor for LRFS, DFS, DMFS, and OS. Thus, the 50% SUVmax method for dose escalation by DP-IMRT is a reasonable recommendation for improving the survival of patients with locoregionally advanced NPC. The therapeutic benefit of a 50%SUVmax threshold regimen for DP-IMRT encourages further exploration in other prospective studies. The present study had several limitations. Our study was limited by its retrospective nature. Although concurrent chemoradiotherapy was the major treatment regimen, induction chemotherapy was administered to 24.3% of patients, which may have influenced the treatment homogeneity. In addition, further follow-up is required to assess the long-term survival of patients with NPC, and more comprehensive PET/CT-guided GTV delineation methods also needed to explore in our future prospective clinical trials to ascertain the most favorable treatment.

## Conclusion

Overall, our results indicated that PET/CT-guided dose escalation IMRT combined with chemotherapy is effective for patients with locoregionally advanced NPC. The 50% SUVmax threshold regimen for DP-IMRT significantly improved survival without any increase in toxicity compared with the visual criteria or SUV2.5 threshold. Further, prospective trials are required to fully investigate the PET/CT-based methods of contouring the tumor to determine an optimal regimen for survival.

## Data Availability Statement

The original contributions presented in the study are included in the article/supplementary material. Further inquiries can be directed to the corresponding author.

## Ethics Statement

The studies involving human participants were reviewed and approved by Ethics Committee of Hunan Cancer Hospital. Written informed consent for participation was not required for this study in accordance with the national legislation and the institutional requirements.

## Author Contributions

Conceived and designed the experiments: FL and HW. Performed the experiments: FL, OY, and YH. Analyzed the data: FL, SF, YC, and OY. Contributed reagents/materials/analysis tools: FL, OY, SF, and YC. Wrote the paper: FL, OY, and YH. All authors contributed to the article and approved the submitted version.

## Funding

This study was supported by grants from Beijing Hope Run Special Fund of Cancer Foundation of China (No. LC2016W05 and LC2016W06), Changsha Science and Technology Bureau (No. kq2004133), and Health Commission of Hunan Province (C2017044). The funders had no role in study design, data collection, and analysis, decision to publish, or preparation of the manuscript.

## Conflict of Interest

The authors declare that the research was conducted in the absence of any commercial or financial relationships that could be construed as a potential conflict of interest.

## Publisher’s Note

All claims expressed in this article are solely those of the authors and do not necessarily represent those of their affiliated organizations, or those of the publisher, the editors and the reviewers. Any product that may be evaluated in this article, or claim that may be made by its manufacturer, is not guaranteed or endorsed by the publisher.
